# The adenylate cyclase-mediated signaling pathway required for regulating siderophore and toxin biosynthesis and pathogenicity in *Alternaria alternata*

**DOI:** 10.3389/ffunb.2026.1766476

**Published:** 2026-02-05

**Authors:** Kai-Chu Huang, Hsin-Yu Lu, Celine Yen Ling Choo, Pei-Ching Wu, Kuang-Ren Chung

**Affiliations:** 1 Department of Plant Pathology, College of Agriculture and Natural Resources, National Chung Hsing University, Taichung, Taiwan; 2 Advanced Plant and Food Crop Biotechnology Center, National Chung Hsing University, Taichung, Taiwan; 3 Department of Medical Laboratory Science and Biotechnology, China Medical University, Taichung, Taiwan

**Keywords:** ACT toxin, adenylate cyclase, autophagy, cAMP, siderophores

## Abstract

The role of cyclic AMP–protein kinase A (PKA) signaling in siderophore-mediated iron uptake and its connection to virulence remains poorly understood in phytopathogenic fungi. Genetic studies demonstrate that the *A. alternata* adenylate cyclase (AaAC) regulates diverse cellular processes, including growth, conidiation, iron homeostasis, autophagy, siderophore biosynthesis, and toxin production. Deletion of *AaAC* results in impaired siderophore secretion, disrupted expression of iron-responsive genes, and a complete loss of ACT toxin biosynthesis, leading to markedly reduced virulence. Transcriptomic analysis under iron-deficient conditions reveals that *AaAC* deletion induces widespread changes in gene expression, notably the downregulation of genes involved in siderophore biosynthesis and ACT toxin production. These findings indicate that AaAC regulates metabolic pathways essential for fungal survival and pathogenicity. Mutants lacking the GTP-binding protein alpha subunit (Gα), the PKA catalytic subunit, or its regulatory subunit also reduce siderophore production. The findings suggest that environmental cues influencing siderophore biosynthesis are transmitted via a signaling cascade from Gα to AaAC and then to PKA. Additionally, AaAC negatively affects autophagy under nutrient-rich conditions. Gene ontology analysis reveals upregulation of autophagy-related genes, suggesting that AaAC may contribute to cellular energy preservation and physiological stability. These results indicate that AaAC is a key integrator of environmental signals, vital for maintaining iron homeostasis, controlling toxin biosynthesis, and driving virulence in *A. alternata*.

## Introduction

The cAMP-dependent signaling pathway is a central regulatory system composed of several key proteins, including G protein-coupled receptors (GPCRs), heterotrimeric G proteins, adenylate cyclase (AC), cAMP-dependent protein kinase A (PKA), and multiple downstream effectors (Huang et al., 2019). This pathway governs essential cellular processes, including growth, differentiation, metabolism, and gene transcription (Daniel et al., 1998; McDonough and Rodriguez, 2011). Within this cascade, AC catalyzes the conversion of ATP into cAMP, which acts as a second messenger to activate PKA and initiate downstream signaling. Beyond its role as a signal generator, AC also functions as a metabolic sensor, integrating cellular energy status with regulatory pathways in eukaryotes (Hanoune et al., 1997). Evolutionarily conserved, AC is found across diverse organisms ranging from bacteria to humans (Casperson et al., 1983).

In fungi, the cAMP–PKA pathway orchestrates vegetative growth, conidiation, stress responses, and virulence (D’Souza and Heitman, 2001; Shimizu and Keller, 2001; Choi and Xu, 2010; Kim et al., 2011; Martin-Urdiroz et al., 2016). Disruption of AC in yeast impairs energy signaling, reduces PKA activity, and leads to severe growth and differentiation defects (Grahl et al., 2015). Similarly, AC inactivation diminishes virulence in multiple pathogenic fungi (Jurick and Rollins, 2007; Kohut et al., 2010; Hu et al., 2014; Wang et al., 2016; Yang et al., 2016; Guo et al., 2019). The pathway also regulates secondary metabolite production, further showing its broad influence on fungal physiology (Mukherjee et al., 2007; García-Rico et al., 2008; Garcia-Martinez et al., 2012; Kohut et al., 2010; Studt et al., 2013).

One of the most successful fungal pathogens, *Alternaria alternata*, infects a wide range of economically important crops, including citrus, apple, pear, rice, strawberry, tomato, broccoli, cauliflower, carrot, and potato, causing significant agricultural losses worldwide. Its pathogenic success largely stems from the production of diverse phytotoxins, particularly host-selective toxins (HSTs), which exhibit specific toxicity and unique mechanisms of action against target plants. Each *A. alternata* pathotype produces a distinct HST, enabling it to infect specific hosts (Hatta et al., 2002; Ito et al., 2004). For example, the tangerine pathotype causes Alternaria brown spot disease on citrus cultivars by producing ACT (*Alternaria citri* tangerine). This toxin induces lipid peroxidation and cell death (Lin et al., 2011). In addition to ACT, virulence depends on the fungal ability to detoxify reactive oxygen species (ROS) (Lin et al., 2009; Yang et al., 2009). Recent studies highlight further contributions of siderophore-mediated iron acquisition, peroxisomes, and autophagy to ROS tolerance and pathogenicity (Chen et al., 2013, 2014; Chung et al., 2020; Choo et al., 2023; Wu et al., 2022, 2023, 2024).

To survive diverse environmental challenges such as oxidative stress and nutrient limitation, fungal pathogens rely on autophagy. This conserved intracellular degradation pathway recycles cellular components and promotes survival under stress (Kraft et al., 2009; Das et al., 2012). Autophagy begins with the formation of a phagophore from ER-associated membranes, which expands into an autophagosome to sequester damaged organelles and proteins for degradation (Gómez-Sánchez et al., 2021). Beyond stress mitigation, autophagy supports fungal growth, conidiation, secondary metabolism, and virulence (Liu et al., 2007; Nguyen et al., 2011; Meng et al., 2025a, b). In *A. alternata*, autophagy is triggered by nitrogen deprivation and oxidative stress, helping eliminate ROS-damaged components and enhancing pathogenicity (Lu et al., 2022; Wu et al., 2022). Moreover, autophagy maintains iron homeostasis: disruption of autophagy-related (Atg) genes reduces siderophore biosynthesis, elevates ROS, and impairs growth under iron deficiency (Wu et al., 2024). Thus, autophagy alleviates oxidative damage and facilitates siderophore production and iron uptake, collectively strengthening virulence. Importantly, iron availability itself modulates autophagy, with both overload and deficiency disrupting autophagic processes and inducing stress (Krishan et al., 2015; Wang et al., 2022).

The cAMP–PKA pathway intersects with autophagy regulation, either promoting or inhibiting it depending on cell type and physiological context (Budovskaya et al., 2004; Grisan et al., 2021). While the role of the cAMP-PKA pathway in general cellular processes is well established, its specific involvement in siderophore biosynthesis remains less explored. In phytopathogenic fungi, siderophores are essential for iron uptake under deficiency, ensuring homeostasis and successful infection. In *A. alternata*, siderophore biosynthesis is regulated by several proteins, such as AaYap1, AaHog1, and AaNox (Chen et al., 2014), as well as AaHapX and AaSreA, which coordinate iron metabolism under fluctuating conditions (Chung et al., 2020; Wu et al., 2022). Recent findings in *Mucor lusitanicus* show that the cAMP–PKA pathway regulates the *rfs* gene, essential for rhizoferrin biosynthesis (Alejandre-Castaneda et al., 2022), suggesting a broader role for this pathway in iron regulation across fungi, including *A. alternata*.

In this study, we demonstrate that the cAMP–PKA signaling pathway, specifically the adenylate cyclase of *A. alternata* (AaAC), is indispensable for regulating both siderophore and ACT toxin biosynthesis. Interestingly, AaAC negatively affects autophagy under nutrient-rich conditions but does not influence oxidative stress resistance. These findings reveal a subtle interplay among signaling pathways, iron regulation, and pathogenicity, providing more profound insight into the molecular mechanisms underlying fungal virulence.

## Materials and methods

### Fungal strains and growth conditions

The wild-type EV-MIL31 strain of *A. alternata* (Fr.) Keissler was isolated from a diseased Minneola tangelo (Citrus x tangelo J.W. Ingram and H.E. Moore) leaf (Lin et al., 2009) and used as a recipient host for transformation and genetic modifications. A ΔΔ*ACTT6* strain with deletions in both *ACTT_6–1* and *ACTT_6-2*, involved in ACT biosynthesis (Ma et al., 2019), was used as a negative control. A Δ*AaNPS6* strain with the deletion of a nonribosomal peptide synthetase (Chen et al., 2013) and a Δ*AaSreA* strain with the deletion of a siderophore repressor (Chung et al., 2020) were used for negative and positive controls, respectively, for siderophore production. All other strains, including Δ*AaGα*, Δ*AaPKA^c^*, and Δ*AaPKA^r^*, were derived from EV-MIL31, and their characteristics are listed in **Supplementary Table S1**. Fungal strains were grown on potato dextrose agar (PDA; Difco, Sparks, MD, U.S.A.) or minimal medium (MM) (Chung et al., 2020) at 28°C for 3 to 5 days. For ACT toxin production, fungi were grown in a modified Richard’s medium (Kohmoto et al., 1991) for 21 days. For conidiation, fungal strains were grown on PDA under constant fluorescent light for 3 to 5 days, and conidia were collected by scraping them off the agar plates with sterile water. For DNA and RNA isolation, fungal strains were cultured in PDB for 2 days. Sensitivity assays were performed by growing fungal strains on PDA or MM amended with an appropriate test compound, and their radial growth was measured. The percentage of growth inhibition compared to the EV-MIL31 strain was calculated as described by Wu et al. (2023).

### Genetic modification of fungi and miscellaneous molecular procedures

The *A. alternata* adenylate cyclase-coding gene (*AaAC*), encoding a protein of 2,111 amino acids, was retrieved from the genome database (Accession No. KAH6839898.1). A split-marker-mediated gene-deletion system (Chung and Lee, 2015) was used to delete a 1,919-bp region of *AaAC*, encompassing the essential adenylate/guanylate cyclase catalytic domain, in the EV-MIL31 strain. Two truncated but overlapping hygromycin-resistant gene (*HYG*) fragments flanked with the *AaAC* sequence (HYg::5’*AaAC* DNA fragment and hYG::3’*AaAC*) were generated by a two-step PCR using gene-specific primers (**Supplementary Figure S1**) and transformed into protoplasts prepared from the EV-MIL31 strain. The CP1 strain was identified by transforming a pCB1532 plasmid carrying a functional copy of *AaAC* and a sulfonylurea-resistant cassette into protoplasts prepared from one of the *AaAC* deletion mutants (Δ*AaAC*_D27). Synthetic GFP, translationally fused to the N-terminus of AaAtg8 (GFP-AaAtg8), was cloned into the pCB1532 vector. The resulting plasmid was then transformed into both the EV-MIL31 and the Δ*AaAC*_D27 strains to study autophagy. Green fluorescence was visualized using a ZOE Fluorescent Cell Imager microscope (Bio-Rad, Hercules, CA, U.S.A.). The GFP-Atg8 proteolysis assay was performed by Western blot analysis as previously described by Lu et al. (2022), based on the notion that the accumulation of free GFP correlates with increasing autophagic activity (Nair et al., 2011). Preparation of fungal protoplasts and transformation were conducted as previously described by Chung et al. (2002). Fungal transformants were recovered from a regeneration medium (RMM) containing 200 µg/ml hygromycin (Roche Applied Science, Indianapolis, IN, USA) or sulfonylurea (10 µg/ml, ChemService Inc., West Chester, PA, U.S.A.) and examined by PCR with various primers, restriction fragment length polymorphism (RFLP), and Southern blot analyses (Wu et al., 2023). Fungal DNA was isolated as described by Choo et al. (2023). The DNA probe was labelled with digoxigenin (DIG)-11-dUTP (Roche Applied Science) by PCR and detected by immunological assays according to the manufacturer’s instructions. Oligonucleotide primers and their sequences used in this study are listed in **Supplementary Table S2**.

### Assays for siderophores, toxin, and virulence

Chrome azurol S (CAS)-containing agar plate assays for siderophore production were conducted as described (Schwyn and Neilands, 1987; Chen et al., 2013). Siderophores and ACT toxin were purified from culture filtrates using Amberlite XAD-16 and Amberlite XAD-2 resins (Sigma-Aldrich, St. Louis, MO, U.S.A.), respectively, and analyzed by thin-layer chromatography (TLC) and high-performance liquid chromatography (HPLC) as described by Wu et al. (2021). Fungal virulence was evaluated by placing agar plugs containing fungal hyphae on detached calamondin (*Citrofortunella mitis*) leaves. Some leaf spots were wounded with a sterilized needle before inoculation. Leaf treated with agar plugs from PDA served as mock controls. All inoculated leaves were placed in a plastic container and incubated at 28°C for 3 days. Each strain was tested on at least five leaves and repeated three times.

### Quantitative RT-PCR and gene expression analyses

Gene expression was assessed using quantitative RT-PCR (qRT-PCR) as described by Wu et al. (2023). RNA was isolated using TRI reagent (Sigma-Aldrich, St. Louis, MO, U.S.A.) and further purified using the Pure LinkTM RNA Mini Kit (Invitrogen, Carlsbad, CA, U.S.A.) according to the manufacturer’s instructions. Complementary DNA (cDNA) was synthesized using the iScript cDNA Synthesis Kit (Bio-Rad) following the manufacturer’s instructions. All qRT-PCR reactions were set up using iQ SYBR Green Supermix (Bio-Rad) and performed in a CFX Connect Real-Time PCR Detection System (Bio-Rad). The comparative Cq (ΔΔCq) method was used to determine the relative gene expression levels, expressed as log2 fold changes. The significant difference was determined by statistical analysis.

### Transcriptomic analysis

Total RNA was extracted from fungal strains cultured in minimal medium (MM) for 2 days under siderophore-inducing conditions. RNA sequencing was carried out by BIOTOOLS (New Taipei City, Taiwan). Libraries were prepared and sequenced on an Illumina platform using paired-end mode, yielding an average of 9 gigabases of reads per sample. Quality assessment of raw sequencing reads was performed with FastQC (Andrews, 2010), and low-quality bases and adapter sequences were trimmed with Trimmomatic (v0.38) (Andrews, 2010; Bolger et al., 2014). Clean reads were aligned to the *A. alternata* strain EV-MIL-31 reference genome (Accession No. JADAKD010000001.1) using HISAT2 (v2.1.0). Gene expression levels were quantified as transcripts per million (TPM). Differential expression analysis was conducted using DESeq2 and edgeR (v1.26.0), with genes exhibiting an absolute log2 fold-change ≥ 2 and an adjusted *p*-value < 0.05 considered significantly differentially expressed. Functional annotation and pathway enrichment analyses were performed using Gene Ontology (GO) and Kyoto Encyclopedia of Genes and Genomes (KEGG) databases.

### Bioinformatics analyses

AaAC orthologs were retrieved from the National Center for Biotechnology Information (NCBI; https://www.ncbi.nlm.nih.gov/). Pairwise sequence comparisons were conducted in CLC Genomic Workbench v9.5.1 (Qiagen, Germantown, MD, U.S.A.) to estimate genetic distances using the Jukes–Cantor model and to calculate percent identity. Multiple sequence alignment of amino acid sequences was conducted using DOG (Domain Graph, v2.0), followed by phylogenetic tree construction using the Maximum Likelihood method implemented in MEGA12. Conserved domains and motifs within the protein sequences were identified using the Conserved Domain Architecture Retrieval Tool (CDART; link) and InterProScan (https://www.ebi.ac.uk/interpro/). Additionally, secondary metabolite gene clusters were predicted using antiSMASH v8.0 (Blin et al., 2025). The analysis was based on the genome assembly of *A. alternata* (ASM1609752v1; GenBank accession: GCA_016097525.1), including GenBank (.gbk) files, nucleotide sequences, and predicted protein sequences.

### Statistical analysis

Unless otherwise specified, all experiments involving multiple replicates were conducted at least twice. Statistical analyses were performed using one-way ANOVA, and significant differences among treatments were identified using Tukey’s HSD *post hoc* test at a significance level of *p* < 0.05.

## Results

### AaAC is required for vegetative growth, conidial development, and iron homeostasis

AaAC, containing a Ras-associating (RA) domain, a leucine-rich repeat (LRR) protein, a protein phosphatase 2C (PP2C), and an adenylate/guanylate cyclase catalytic (CYCc) domain, is highly conserved among fungi, showing ~90% sequence similarity (**Supplementary Figure S2**). Using the split-marker approach, two fungal strains, Δ*AaAC*_D27 and Δ*AaAC*_D30, were identified as lacking the *AaAC* gene. Both mutants exhibited reduced growth under nutrient-rich (PDA) and nutrient-poor (MM) conditions (**Supplementary Figure S3**). When cultured on PDA and MM media, Δ*AaAC* exhibited growth reductions of approximately 50% and 70%, respectively, relative to the wild-type (WT) and the CP1 complementation strains. Δ*AaAC* exhibited deformed hyphae characterized by globular swellings. In addition, conidial production was severely impaired in both mutants. Reintroduction of the *AaAC* gene into the Δ*AaAC*_D27 strain generated the CP1 strain, which restored conidial formation with size and morphology comparable to those of the WT strain. Δ*AaAC* exhibited similar growth inhibition on PDA and PDA supplemented with FeSO_4_, FeCl_3_, or bathophenanthroline disulfonic acid (BPS), an iron chelator (**Supplementary Figure S4**). In contrast, when assayed on MM, Δ*AaAC* displayed heightened sensitivity to FeSO_4_ and FeCl_3_, while increasing concentrations of BPS progressively mitigated this sensitivity. Δ*AaAC* showed comparable growth inhibition on PDA and on PDA supplemented with H_2_O_2_, but exhibited reduced sensitivity to Congo red at higher concentrations.

### AaAC is involved in siderophore biosynthesis

**Figure 1:**
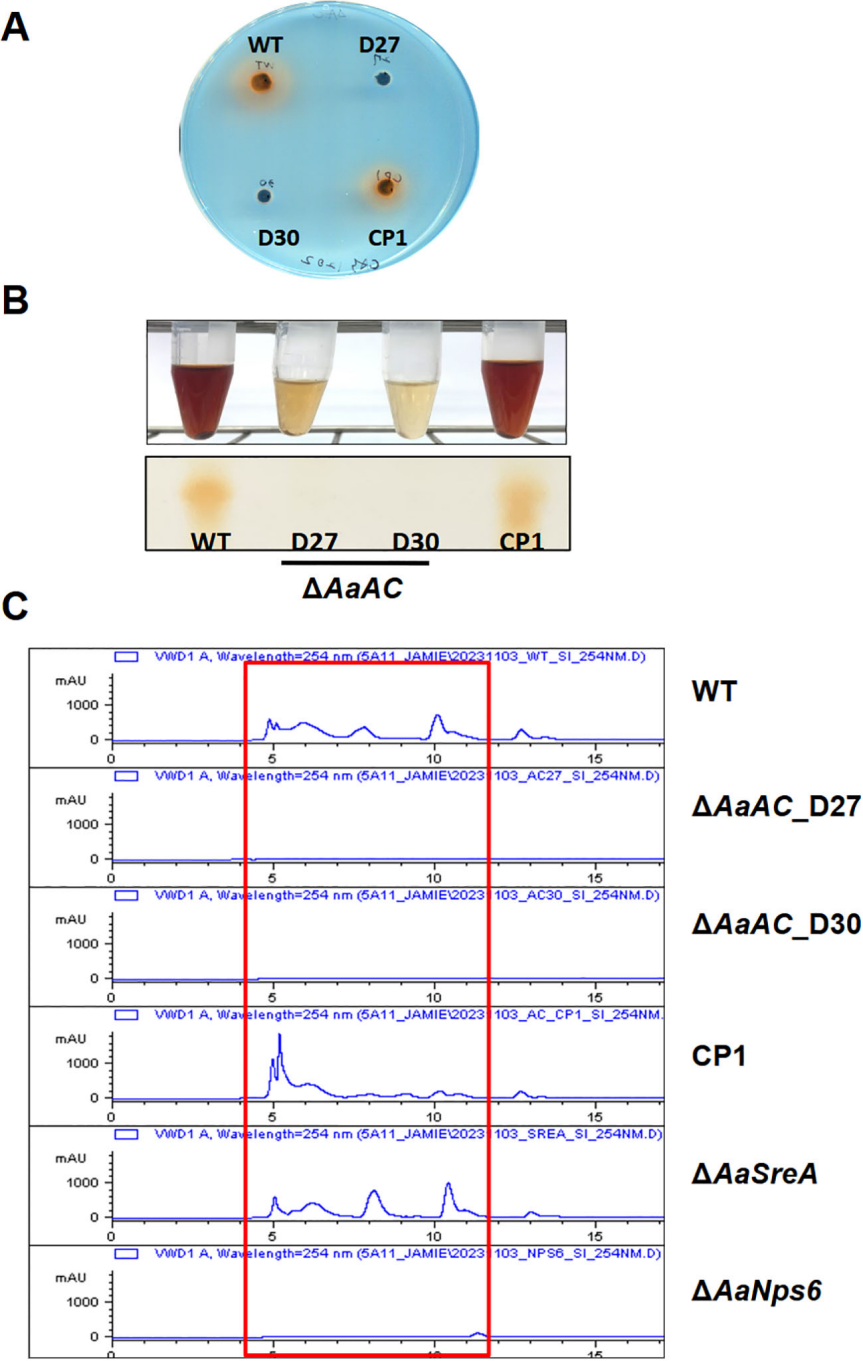
AaAC is required for siderophore production. **(A)** CAS plate assays reveal distinct orange halos in WT and CP1 strains, indicative of siderophore secretion, whereas Δ*AaAC* strains (D27 and D30) lack halos, suggesting that AaAC is essential for this process. **(B)** The FeCl_3_ reactivity assay and TLC analysis confirm these observations. **(C)** HPLC analysis identifies multiple peaks in WT and CP1 samples, as well as in Δ*AaSreA* (a positive control defective in the siderophore suppressor gene). No peaks are detected in Δ*AaAC* or Δ*AaNps6* strains, the latter lacking a functional nonribosomal peptide synthetase gene (negative control).

**Figure 2:**
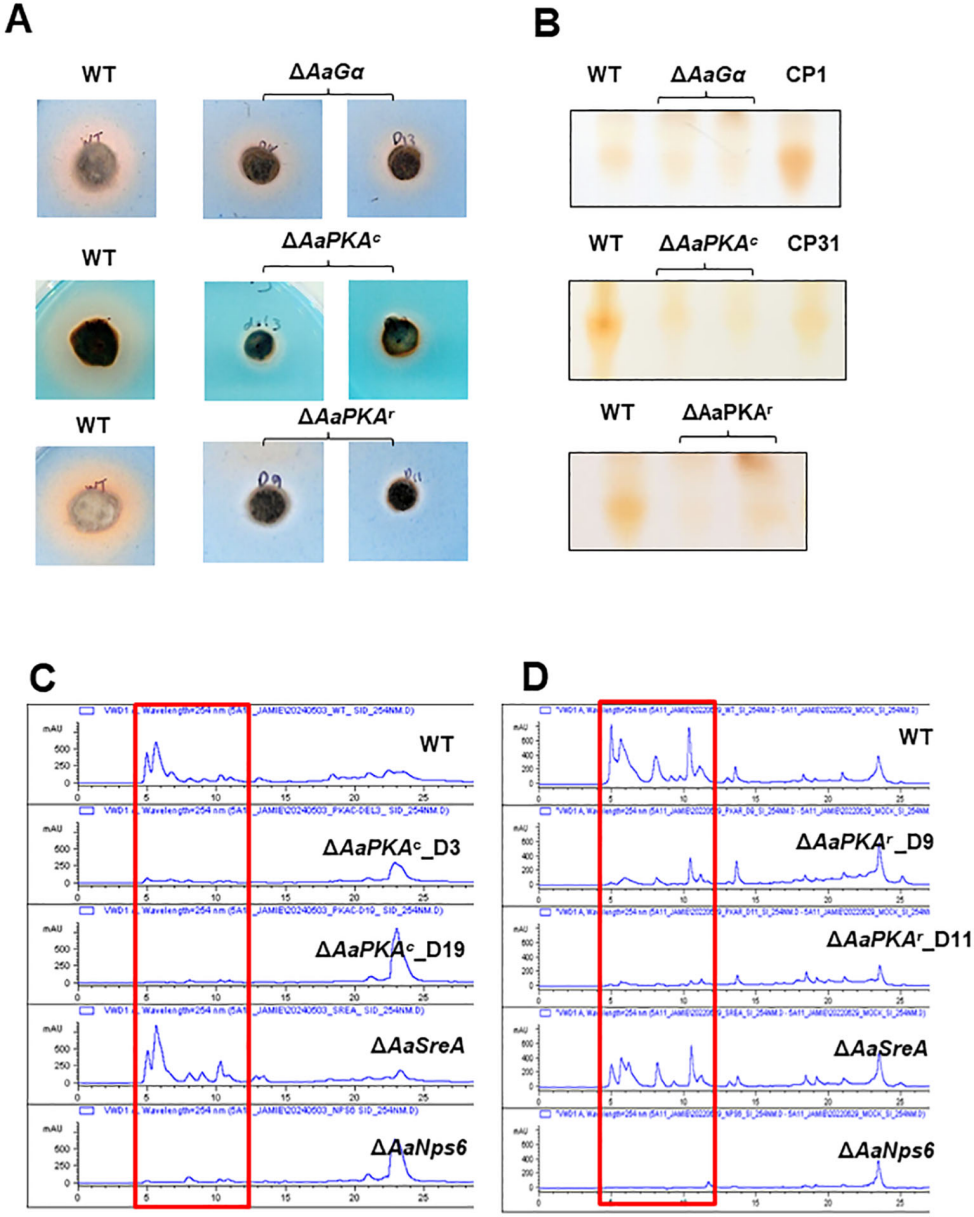
Gα, PKAc, and PKAr contribute to siderophore production. **(A)** CAS plate assays show smaller orange halos in mutant strains compared to WT. **(B)** TLC analysis confirms these observations. **(C, D)** HPLC validates reduced siderophore production in the mutants.

CAS plate assays were initially performed to assess the ability of fungal strains to produce siderophores. The WT and CP1 strains formed distinct orange halos on CAS plates (**Figure 1A**), signifying active siderophore production. In contrast, Δ*AaAC* produced no visible halos. The production of siderophores was confirmed further by purifying them from culture filtrates. Siderophores purified from the culture filtrates of the WT and CP1 strains reacted with FeCl_3_ to produce a dark red color, in stark contrast to the light brown hue observed in the filtrates from Δ*AaAC*. TLC analysis of purified siderophores revealed distinct dark orange bands at *R_f_* 0.73 in the WT and CP1 samples. In contrast, no such bands were detected in the Δ*AaAC* samples (**Figure 1B**). HPLC analysis of purified siderophores from the WT and CP1 strains revealed multiple peaks between 5 and 13 min (retention time), with a prominent peak at 5 min (**Figure 1C**). Similar peaks were also observed in samples from the Δ*AaSreA* strain, which is defective in the siderophore suppressor gene. No corresponding peak was detected in the Δ*AaAC* samples, nor in the Δ*AaNps6* strain, which lacks a functional nonribosomal peptide synthetase. CAS, TLC, and HPLC assays revealed that fungal strains lacking the GTP-binding protein alpha subunit (Gα), the protein kinase A catalytic subunit (PKA^c^), or its regulatory subunit (PKA^r^) exhibited reduced siderophore production (**Figure 2**).

### AaAC regulates the expression of genes involved in siderophore production

**Figure 3:**
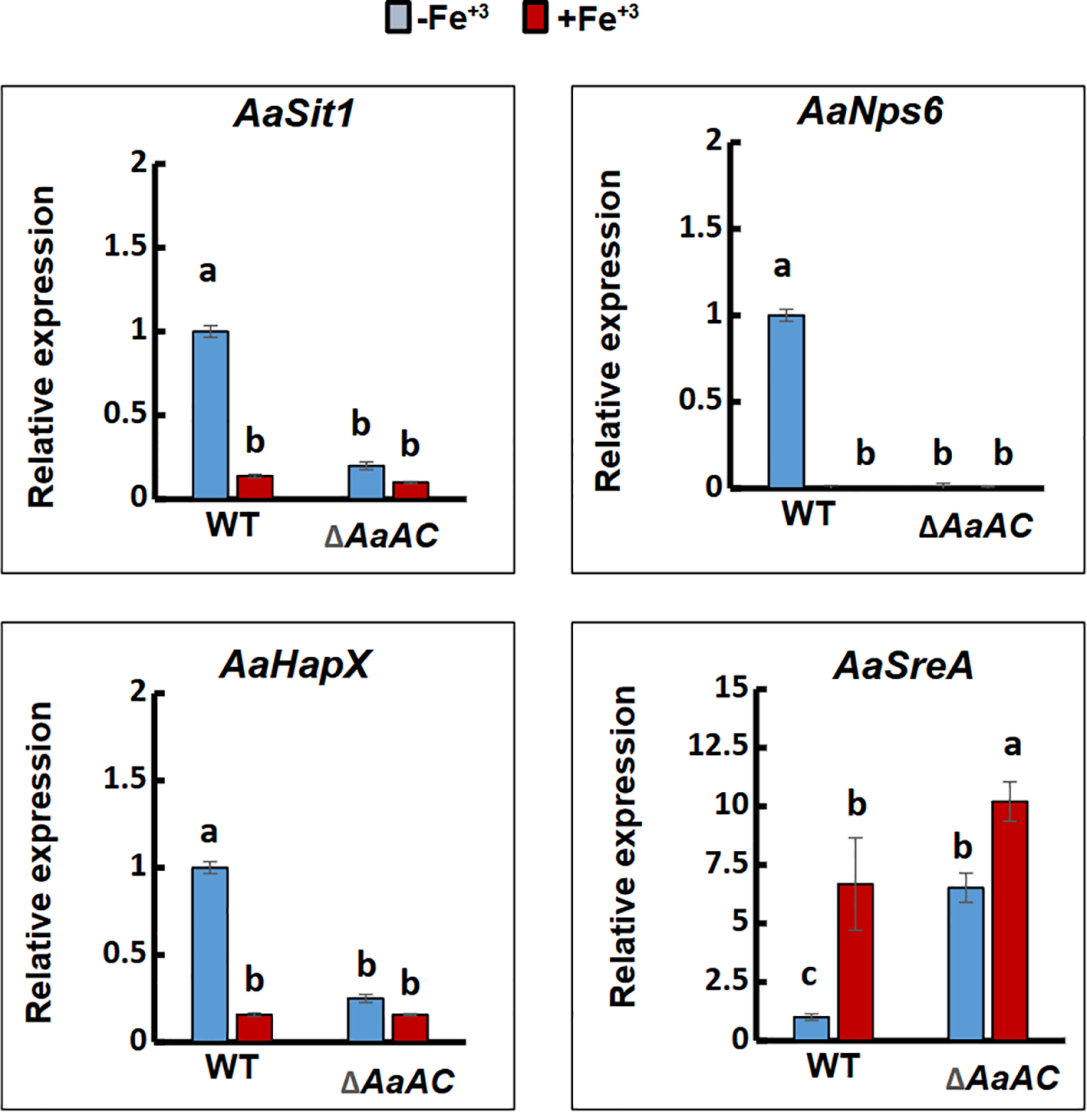
Quantitative real-time PCR analysis shows that *AaAC* deletion led to significant downregulation of *AaSit1* (siderophore transporter), *AaNps6* (nonribosomal peptide synthetase), and *AaHapX* (bZIP transcription factor). In contrast, *AaSreA* (repressor of siderophore biosynthesis) is upregulated. Means followed by the same letters are not significantly different, *p* < 0.05.

Quantitative real-time PCR analysis revealed that under iron-deficient conditions (MM), the expression levels of three siderophore production-related genes: *AaSit1* (Accession No. KAH8623772), encoding a siderophore transporter; *AaNps6* (Accession No. KAH8635963), encoding a nonribosomal peptide synthetase (NRPS); and *AaHapX* (Accession No. KAH8641358), encoding a basic leucine zipper (bZIP) transcriptional regulator, were significantly downregulated in the Δ*AaAC*_27 mutant strain (**Figure 3**). In contrast, the expression of the *AaSreA* gene (Accession No. KAH8622335) (**Figure 3**), which encodes a siderophore repressor, was upregulated in Δ*AaAC* compared to the WT strain grown in MM. This upregulation was further enhanced under high iron conditions.

### AaAC is involved in the biosynthesis of host-selective toxins

**Figure 4:**
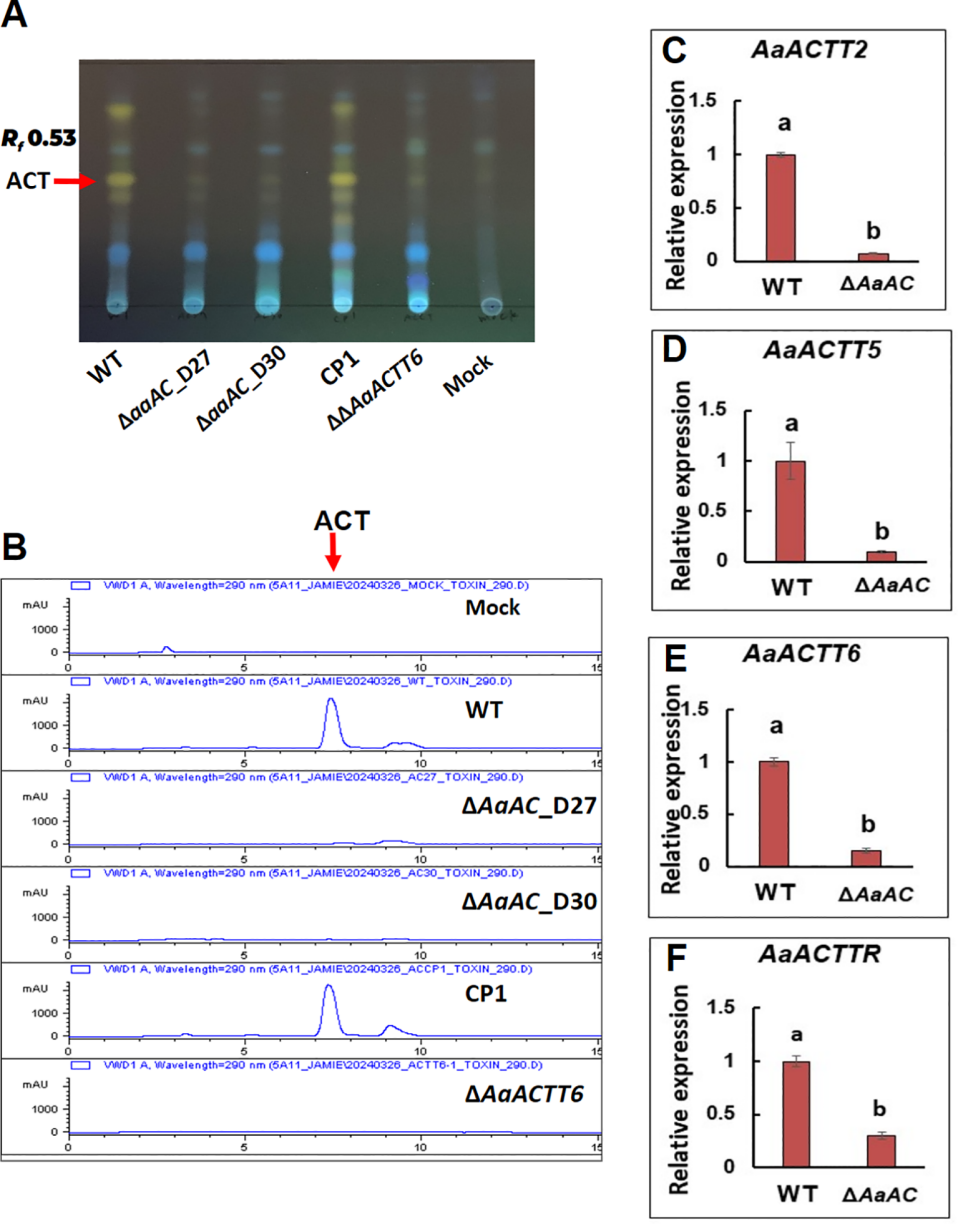
AaAC is involved in the biosynthesis of the ACT toxin. **(A)** TLC analysis of culture filtrates reveals a distinct band at *R_f_* 0.53 in WT and CP1, but only faintly in ΔAaAC strains. **(B)** HPLC confirms these findings, showing a prominent peak at 7.5 min in WT and CP1, but absent in the mutants. **(C–F)** Quantitative real-time PCR further demonstrates that AaAC regulates the expression of *AaACTT2*, *AaACTT5*, *AaACTT6*, and *AaACTTR*, all of which are implicated in ACT biosynthesis.

TLC analysis of purified toxins revealed a distinct band at *R_f_* 0.53 in the WT and CP1 samples. In contrast, the band was barely detectable in samples purified from the Δ*AaAC* and the Δ*AaACTT6* strains (**Figure 4A**). No corresponding band was observed in the control sample derived from medium alone. HPLC analysis of toxins from the WT and CP1 strains showed a prominent peak at a retention time of 7.5 min (**Figure 4B**), which was absent in the samples from Δ*AaAC* and Δ*AaACTT6*. Furthermore, quantitative real-time PCR analysis under iron-deficient conditions demonstrated significant downregulation of four ACT biosynthesis-related genes: *ACTT2* (Accession No. KAH8621102), *ACTT5* (KAH8621105), *ACTT6* (KAH8621103), and *ACTTR* (KAH8621124) in the Δ*AaAC* strain (**Figures 4C–F**).

### AaAC is required for fungal pathogenicity

**Figure 5:**
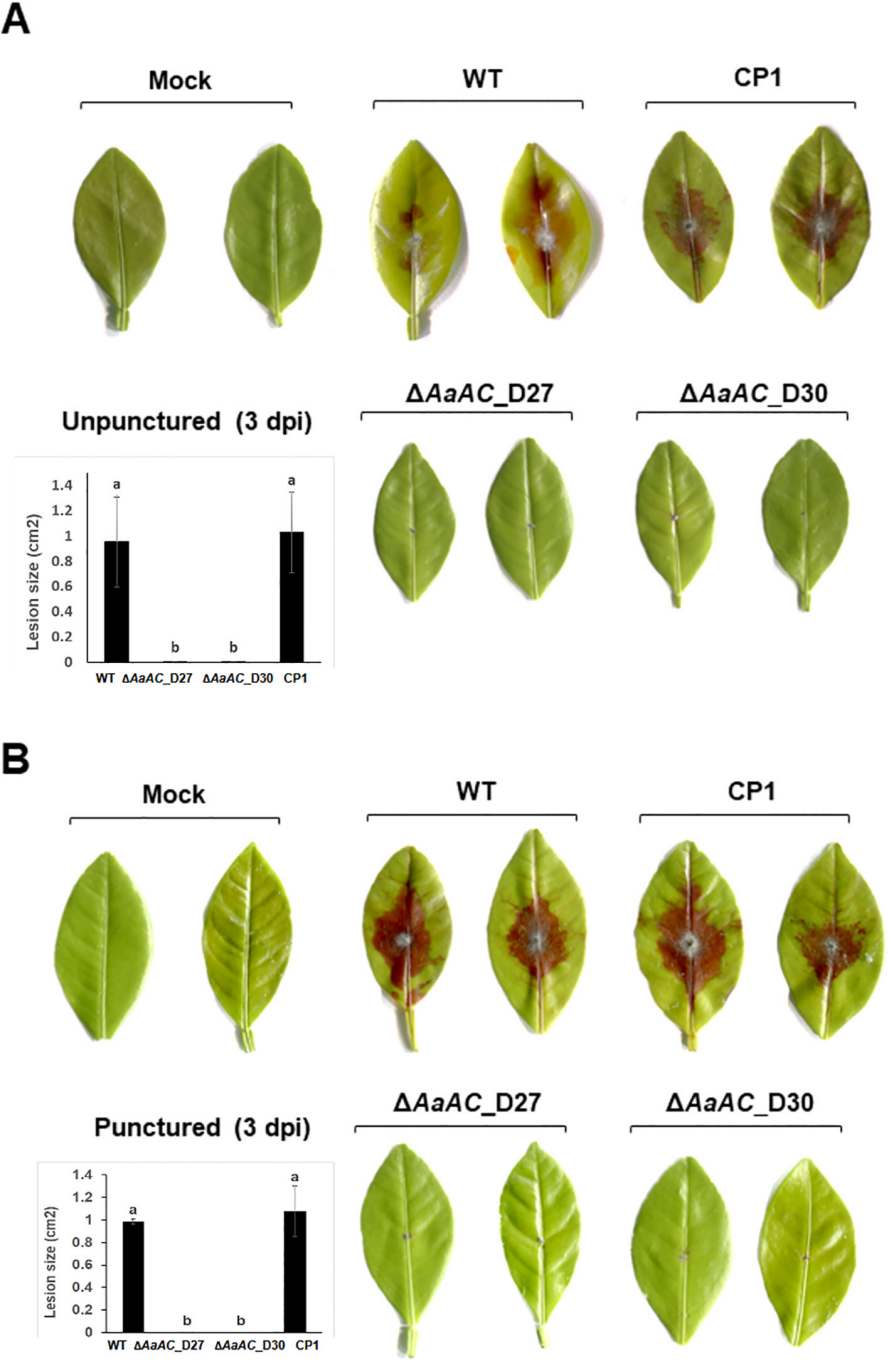
AaAC is required for *A. alternata* virulence. **(A)** Detached calamondin leaves without prior wounding develop necrotic lesions after inoculation with the WT and CP1 strains, but not with the D27 or D30 strains, at 3 days post-inoculation (dpi). **(B)** Similar results were observed on leaves wounded with a fine needle before inoculation. Fungal inoculation was performed by placing agar plugs containing hyphae onto leaves; PDA agar plugs served as mock controls. Each strain was tested on at least five leaves and repeated three times. The area of necrotic lesions was measured using Image J. The significance of the data was analyzed via the Turkey’s HSD *post-hoc* test. Means followed by the different letters are significantly different, *p* < 0.05.

Fungal pathogenicity was assessed using detached calamondin leaves inoculated with agar plugs containing fungal hyphae, as the Δ*AaAC*_D27 and Δ*AaAC*_D30 strains were unable to produce conidia. The WT and CP1 strains induced necrotic lesions on all test leaves, regardless of wounding (**Figure 5**). In contrast, no lesions were observed on leaves inoculated with Δ*AaAC*_D27 or Δ*AaAC*_D30, nor on those treated with agar plugs alone. Pre-inoculation wounding did not promote lesion formation in the Δ*AaAC*_D27 and Δ*AaAC*_D30 strains.

### Transcriptomic analyses confirm the regulatory role of AaAC in the biosynthesis of siderophores and ACT toxin

**Figure 6:**
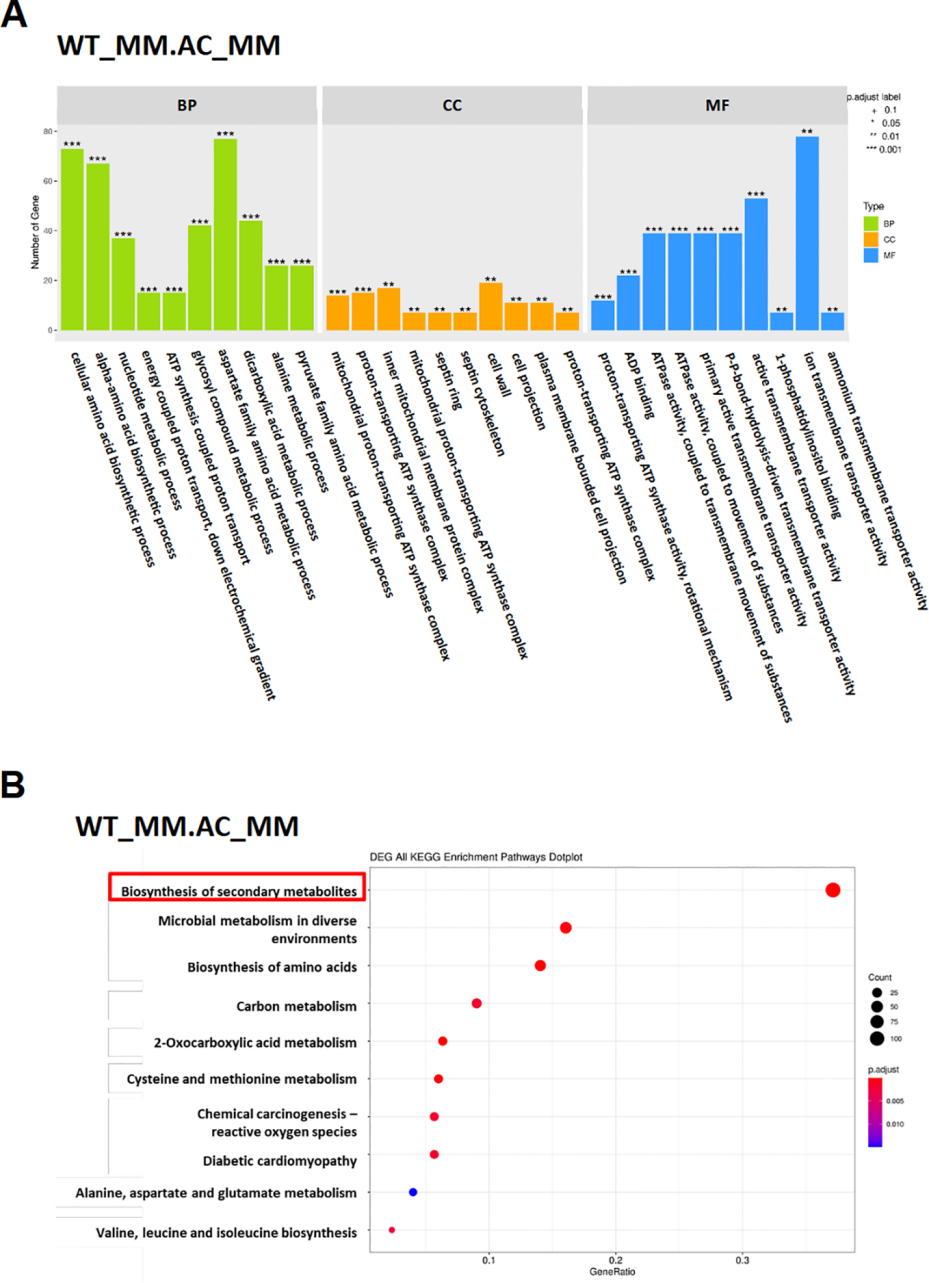
Transcriptome analysis of differentially expressed genes (DEGs) in Δ*AaAC*. **(A)** Gene Ontology (GO) enrichment analysis of DEGs in Δ*AaAC* compared to WT. **(B)** Scatter plots of KEGG pathway enrichment based on statistical analysis of DEGs in Δ*AaAC*. Only the top 10 enriched pathway terms are shown.

RNA-seq analysis was conducted to compare whole-genome expression profiles between the Δ*AaAC*_D27 mutant and the WT strain. Loss of AaAC resulted in a broad spectrum of differentially expressed genes (DEGs), primarily associated with the “cellular amino acid biosynthetic process,” “aspartate family amino acid metabolic process,” and “transmembrane transporter activity” under iron-deficient conditions (**Figure 6A**). Additionally, many DEGs were enriched in secondary metabolite biosynthesis (**Figure 6B**). Gene ontology analysis further revealed significant downregulation of several genes putatively involved in siderophore biosynthesis and transport in Δ*AaAC*_D27. These included genes encoding nonribosomal peptide synthetase 4, siderophore iron transporter, ferric reductase, siderochrome-iron transporter-like protein Sit1, ferric-chelate reductase, iron transport multicopper oxidase, L-ornithine 5-monooxygenase, ABC-type Fe³^+^ transport system, siderophore iron transporter MirC, iron-sulfur cluster assembly protein-like protein 1, and monothiol glutaredoxin-like protein 4 (**Supplementary Table S3**). In contrast, the gene encoding ornithine aminotransferase (IG631_12487), involved in the conversion of ornithine to proline, and the gene encoding arginase (IG631_11003), engaged in the conversion of arginine to ornithine (Beckmann et al., 2013), were significantly upregulated in Δ*AaAC*_D27.

**Figure 7:**
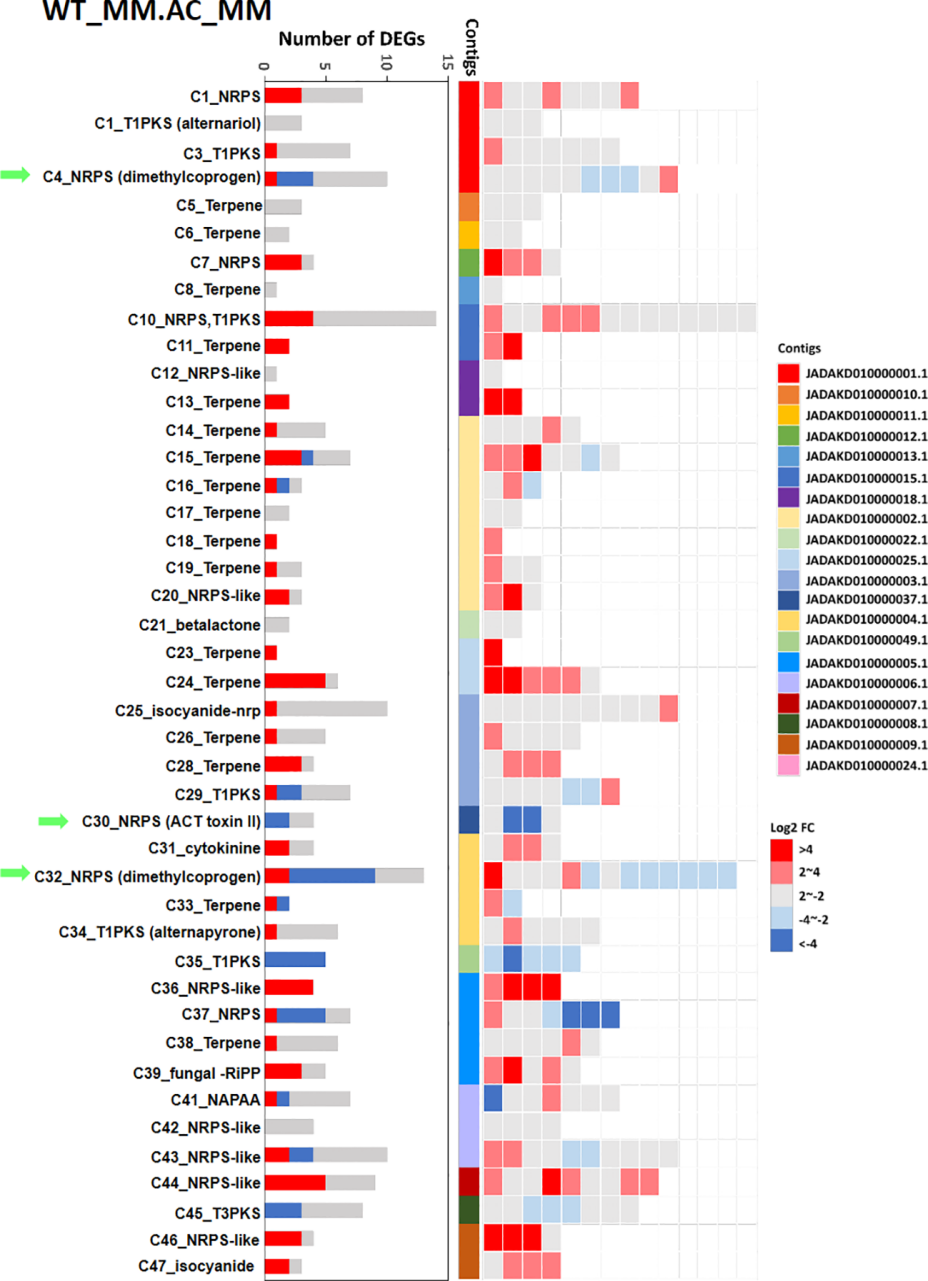
Differential expression of gene clusters associated with the secondary metabolite biosynthesis between the Δ*AaAC* and the WT strains. NRPS, nonribosomal peptide synthetase; T1PKS, type I polyketide synthase; Terpene, terpene synthase; NAPAA, Non-alpha poly-amino acids like e-Polylysin; RiPP, Other unspecified ribosomally synthesized and post-translationally modified peptide product.

Gene ontology analysis also revealed downregulation of several genes putatively involved in ACT toxin biosynthesis in Δ*AaAC*_D27. These included genes encoding *S*-Adenosyl-L-methionine (SAM), hydroxymethylglutaryl-CoA hydrolase (HMGH), hydroxymethylglutaryl-CoA synthase (HMGS), enoyl-CoA hydrolase, cytochrome P450 monooxygenase (P450 or CYP), non-ribosomal peptide synthetase (NRPS), and enoyl-CoA hydratase (ECH) (**Supplementary Table S3**). To investigate the role of AaAC in regulating virulence via secondary metabolite production, the transcriptional regulation of biosynthetic gene clusters in *A. alternata* was predicted using antiSMASH 8.0 (**Figure 7**). The analysis revealed that AaAC broadly influenced the expression of multiple gene clusters involved in secondary metabolite biosynthesis. Notably, the Δ*AaAC* mutant showed strong downregulation of genes in clusters 4 (dimethylcoprogen), 32 (dimethylcoprogen), and 30 (ACT toxin).

### AaAC suppresses autophagy under nutrient-rich conditions

**Figure 8:**
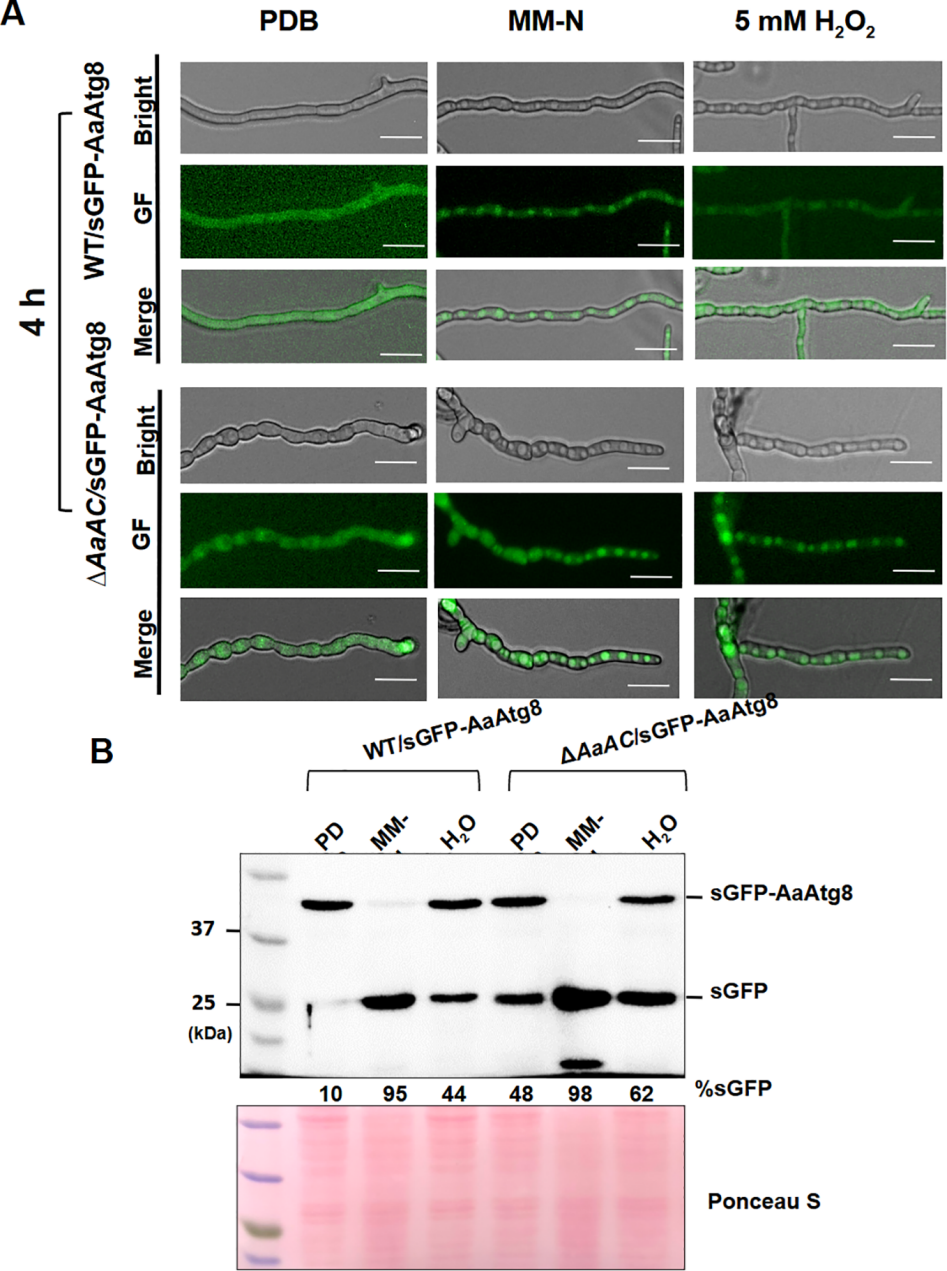
AaAC suppresses autophagy under nutrient-rich conditions. **(A)** Fluorescence microscopy of WT and Δ*AaAC* hyphae expressing sGFP-AaAtg8, cultured in PDB, MM-N, or under oxidative stress (5 mM H_2_O_2_). Vacuolar green fluorescence indicates autophagy. **(B)** Western blot of protein extracts from WT and Δ*AaAC* strains. Free sGFP intensity relative to total sGFP-AaAtg8 was quantified with ImageJ to assess autophagic flux. Ponceau S staining was conducted to ensure equal loading. Bar = 25 µm.

The WT and Δ*AaAC*_D27 strains expressing synthetic GFP translationally fused to the N-terminus of AaAtg8 (GFP-AaAtg8) were employed to monitor autophagosome formation under various conditions. Fluorescence microscopy revealed uniform green fluorescence in the WT hyphae expressing GFP-AaAtg8 when cultured in nutrient-rich medium. In contrast, hyphae grown in MM lacking nitrogen (MM-N) or in PDB supplemented with 5 mM H_2_O_2_ exhibited punctate green fluorescence, indicative of autophagy activation (**Figure 8A**). The Δ*AaAC*_D27 strain expressing GFP-AaAtg8 exhibited punctate green fluorescence in hyphae under all tested conditions, including PDB, MM-N, and PDB supplemented with H_2_O_2_. A GFP-Atg8 proteolysis assay was performed by Western blot analysis to assess autophagic flux (**Figure 8B**). In the WT strain expressing GFP-AaAtg8, approximately 10% free GFP was detected when cultured in PDB, and upon shifting to MM-N and PDB supplemented with H_2_O_2_, the levels of free GFP increased to 95% and 44%, respectively. In contrast, the Δ*AaAC*_D27 strain expressing GFP-AaAtg8 exhibited elevated levels of free GFP under all conditions, with 48% in PDB, 98% in MM-N, and 62% in PDB supplemented with H_2_O_2_. Gene ontology analysis further revealed significant downregulation of several autophagy-related genes. These included: Autophagy-related protein 13, Autophagy-related protein 3, and autophagocytosis protein (**Supplementary Table S3**).

## Discussion

The cAMP-PKA signaling pathway plays an important, evolutionarily conserved role in regulating key cellular processes in numerous fungi (Martin-Urdiroz et al., 2016). Adenylate cyclase synthesizes cAMP and serves as a key component of the G protein–cAMP–PKA signaling pathway (McDonough and Rodriguez, 2011). In this study, we demonstrate that *A. alternata* AaAC functions as a master regulatory protein essential for its physiology, metabolism, and pathogenicity. It governs a wide array of cellular processes, including growth and development, maintenance of cell wall integrity, metabolic adaptation under stress conditions, suppression of autophagy, regulation of iron homeostasis, siderophore biosynthesis, and toxin production.

Although AC is conserved in fungi, its role in conidia formation could vary. AaAC is required for conidial formation. In contrast, an AC homolog in the citrus green mold pathogen *Penicillium digitatum* negatively affects conidial formation (Wang et al., 2016). AaAC has intricate interactions with iron metabolism, as AaAC-deficient mutants exhibit increased sensitivity to iron salts, suggesting a role in regulating iron levels under nutrient stress. Interestingly, BPS treatment alleviates this sensitivity, suggesting iron overload, rather than deficiency, exacerbates the growth defect in the absence of AaAC. Although AaAC appears non-essential for oxidative stress resistance, altered Congo red responses imply changes in cell wall dynamics. AaAC may function as a context-dependent modulator, possibly integrating nutrient and stress signals to maintain cellular homeostasis.

AaAC serves as a central regulator of siderophore biosynthesis and iron homeostasis, as evidenced by the absence of detectable siderophore activity in Δ*AaAC*. The lack of halos in Δ*AaAC* suggests a complete loss of siderophore production, implicating AaAC as essential for this process. The FeCl_3_ reactivity assay offers a simple yet effective chemical validation of siderophore presence. The distinct dark red coloration observed in WT and CP1 filtrates, in stark contrast to the light brown hue of Δ*AaAC*, supports findings from the CAS plate assay. Further confirmation is provided by TLC and HPLC analyses, in which bands and peaks detected in WT and CP1 samples but absent in Δ*AaAC* appear to correspond to specific siderophore compounds whose synthesis depends on AaAC function. Furthermore, diminished siderophore production in strains deficient in Gα, the PKA catalytic subunit, or the PKA regulatory subunit indicates that siderophore biosynthesis is governed by a broader signaling network involving the G-protein- and cAMP-dependent pathway. The cAMP-PKA pathway is also involved in rhizoferrin siderophore secretion in *Mucor lusitanicus* (Alejandre-Castaneda et al., 2022), further supporting its role in siderophore biosynthesis.

The quantitative real-time PCR analysis reveals that deletion of AaAC disrupts the regulatory network governing iron homeostasis. Under iron-deficient conditions, there is a marked downregulation of genes encoding AaSit1, AaNps6, and AaHapX, key components of the iron acquisition and regulatory machinery. This indicates that deletion of AaAC impairs the fungal response to iron scarcity. Δ*AaAC* likely fails to properly sense or adapt to iron limitation, potentially compromising its survival and virulence in iron-depleted environments. In contrast, the observed upregulation of the gene encoding AaSreA, a known repressor of siderophore biosynthesis (Chung et al., 2020), further supports the hypothesis of a dysregulated iron response.

AaAC is not only involved in toxin biosynthesis but may also play a regulatory role in coordinating the expression of toxin-related genes. The TLC and HPLC analyses show that AaAC is necessary for the synthesis or accumulation of ACT toxins. The significant downregulation of toxin biosynthesis-related genes, *ACTT2*, *ACTT5*, *ACTT6*, and *ACTTR*, in Δ*AaAC* under iron-deficient conditions suggests that AaAC acts upstream in a regulatory cascade controlling their expression. Iron limitation is a known environmental cue that influences secondary metabolism (Canessa and Larrondo, 2013; Haas, 2015), and the observed transcriptional changes imply that AaAC may integrate ecological signals to modulate toxin production in response to such stress. Disruption of *AaAC* results in the loss of detectable toxin products and the downregulation of key biosynthetic genes, thereby reducing virulence. This signaling pathway likely influences the function of the PKA^r^ regulatory subunit rather than the PKA^c^ catalytic subunit, as deletion of *PKAr*, but not *PKAc*, significantly impairs *A. alternata* virulence (Tsai et al., 2013). Furthermore, the Gα subunit appears to play no role in toxin biosynthesis or virulence (Wang et al., 2010). This suggests that environmental signals regulating ACT toxin biosynthesis are not transduced through the Gα subunit. Nevertheless, AaAC remains a critical component in ACT toxin biosynthesis in *A. alternata*. A recent study by Yang et al. (2016) also shows that AC is involved in both aflatoxin biosynthesis and *Aspergillus flavus* virulence.

The inability of Δ*AaAC* to induce necrotic lesions strongly implicates AaAC as a critical determinant of pathogenicity in a detached calamondin leaf assay. Since Δ*AaAC* failed to produce conidia, pathogenicity was assessed by hyphal inoculation. This approach bypasses spore germination and directly tests the ability of vegetative mycelia to initiate infection. Pre-inoculation wounding did not restore lesion formation in Δ*AaAC*, indicating that the loss of pathogenicity is not due to impaired entry through intact plant surfaces. The failure of hyphae from Δ*AaAC* to cause disease suggests that AaAC may regulate factors beyond conidiation, including toxin production, host penetration, and iron acquisition. Thus, AaAC is involved in active virulence mechanisms rather than passive colonization.

The transcriptomic analysis confirms the central roles of AaAC in regulating siderophore biosynthesis and of the ACT toxin. The RNA-seq comparison between Δ*AaAC* and WT under iron-deficient conditions reveals broad, significant changes in gene expression, highlighting the profound influence of AaAC on cellular metabolism and secondary metabolite production. The enrichment of DEGs in many biosynthetic and metabolic pathways suggests that AaAC plays a pivotal role in coordinating metabolic responses to iron limitation. These pathways are critical for maintaining cellular homeostasis and supporting the biosynthetic demands of secondary metabolite production, particularly under nutrient-stress conditions. A key finding is the downregulation of multiple genes involved in siderophore biosynthesis and iron acquisition in Δ*AaAC*. The repression of these genes further indicates that AaAC is a positive regulator of siderophore-mediated iron uptake, which is a crucial function for fungal survival and virulence in iron-limited host environments. The upregulation of ornithine aminotransferase, which converts ornithine to proline, and arginase, which converts arginine to ornithine (Beckmann et al., 2013), suggests a compensatory metabolic shift. Ornithine is a key precursor in siderophore biosynthesis (Hissen et al., 2005). The deletion of AaAC may reroute nitrogen flux toward proline biosynthesis or to maintain ornithine pools in the absence of siderophore production. The redirection of metabolic flow could reduce intracellular ornithine availability for siderophore biosynthesis, thereby contributing to the observed reduction in siderophore biosynthesis. This metabolic reprogramming may indicate that Δ*AaAC* attempts to adapt to the loss of AaAC function.

The transcriptomic analysis also demonstrates that AaAC is essential for the expression of genes involved in ACT toxin biosynthesis. The downregulation of genes encoding key enzymes such as SAM synthase, HMGS, HMGH, NRPS, and cytochrome P450 monooxygenases in Δ*AaAC* suggests that AaAC is a master regulator of this virulence-associated secondary metabolite pathway. Given the role of the ACT toxin in host tissue necrosis and disease progression, downregulation of the biosynthetic genes in the mutant strain may significantly attenuate pathogenicity. The antiSMASH-based prediction of biosynthetic gene clusters further supports the broad regulatory scope of AaAC. The observed downregulation of clusters 4 (NRPS), 32 (dimethylcoprogen), and 30 (ACT toxin) in Δ*AaAC* indicates that AaAC functions as a global transcriptional regulator of secondary metabolism. This suggests that AaAC may function upstream of multiple biosynthetic pathways, potentially through direct transcriptional activation or by modulating other regulatory elements.

The reduction in siderophore and toxin production observed in Δ*AaAC* may result from metabolic disruptions triggered by impaired cAMP signaling. In the absence of adenylate cyclase, intracellular cAMP levels may decline, thereby reducing protein kinase A activity and potentially compromising cellular energy sensing and metabolic homeostasis (Dai et al., 2025). This imbalance may impair mitochondrial function and diminish tricarboxylic acid cycle flux, thereby limiting the availability of key metabolic intermediates such as acetyl-CoA. As acetyl-CoA is a critical precursor in nonribosomal peptide synthesis (Gründlinger et al., 2013), which is required for both siderophore and toxin biosynthesis, its reduced availability could directly impact metabolite output. Thus, the defect in siderophore production in Δ*AaAC* likely arises from a combination of transcriptional repression and a shortage of essential biosynthetic substrates, both of which contribute to metabolic dysfunction.

Beyond its roles in regulating siderophores and the ACT toxin, AaAC acts as an autophagy suppressor under nutrient-rich conditions. Fluorescence microscopy reveals only low levels of autophagy in the WT strain cultured in nutrient-rich PDB medium. The formation of autophagosomes and activation of autophagy are induced by nitrogen starvation or oxidative stress, consistent with known fungal survival strategies under adverse environmental conditions (Palmer et al., 2008; Bartoszewska and Kiel, 2010). Strikingly, Δ*AaAC* exerts autophagy across all tested conditions, including nutrient-rich PDB. This constitutive autophagy phenotype indicates that AaAC is required to repress autophagosome formation under nutrient-sufficient conditions, and its absence leads to deregulated autophagy regardless of environmental cues. The Western blot analysis of GFP-Atg8 proteolysis further substantiates the microscopy findings. The suppression of autophagy by AaAC under favorable growth conditions may serve to conserve cellular resources and maintain metabolic efficiency. Autophagy is energetically costly, and its unnecessary activation could disrupt cellular homeostasis. Therefore, AaAC likely contributes to the fine-tuning of autophagic responses, ensuring that this catabolic process is only engaged when necessary. Although autophagy is typically regarded as a mechanism that promotes cellular survival under stress, excessive or dysregulated autophagy could lead to cell death (Kraft et al., 2009; Bartoszewska and Kiel, 2011; Das et al., 2012). Deregulated autophagy in Δ*AaAC* may influence its development, stress tolerance, and virulence.

## Conclusion

Our findings establish AaAC as a central regulator within the cAMP–PKA signaling pathway, orchestrating diverse physiological and pathogenic processes in *A. alternata*. Beyond its conserved role in fungal growth and development, AaAC integrates nutrient and stress signals to maintain cellular homeostasis, particularly by regulating iron metabolism, siderophore biosynthesis, and toxin production. Its deletion disrupts metabolic balance, represses key biosynthetic genes, abolishes siderophore and toxin output, and severely attenuates pathogenicity. AaAC is also a key suppressor of autophagy under nutrient-sufficient conditions, thereby preventing unnecessary activation of this energetically demanding process. These results highlight AaAC as a key hub that coordinates environmental stress responses with secondary metabolism, underscoring its essential role in fungal virulence and survival.

## Data Availability

The original contributions presented in the study are publicly available. This data can be found here: NCBI database, accession numbers KAH6839898.1 (AaAc), KAH8623772 (AaSit1), KAH8623772 (AaNps6), KAH8641358 (AaHapX), and KAH8622335 (AaSreA).
