# Correction: The adenylate cyclase-mediated signaling pathway required for regulating siderophore and toxin biosynthesis and pathogenicity in *Alternaria alternata*

**DOI:** 10.3389/ffunb.2026.1884384

**Published:** 2026-07-02

**Authors:** Kai-Chu Huang, Hsin-Yu Lu, Celine Yen Ling Choo, Pei-Ching Wu, Kuang-Ren Chung

**Affiliations:** 1 Department of Plant Pathology, College of Agriculture and Natural Resources, National Chung Hsing University, Taichung, Taiwan; 2 Advanced Plant and Food Crop Biotechnology Center, National Chung Hsing University, Taichung, Taiwan; 3 Department of Medical Laboratory Science and Biotechnology, China Medical University, Taichung, Taiwan

**Keywords:** ACT toxin, adenylate cyclase, autophagy, cAMP, siderophores

There was a mistake in **Figure 2B** as published. The discrepancy appears to have resulted from a processing miscropping, which inadvertently altered the presentation of the data. The corrected **Figure 2** appears below.

**Figure 2:**
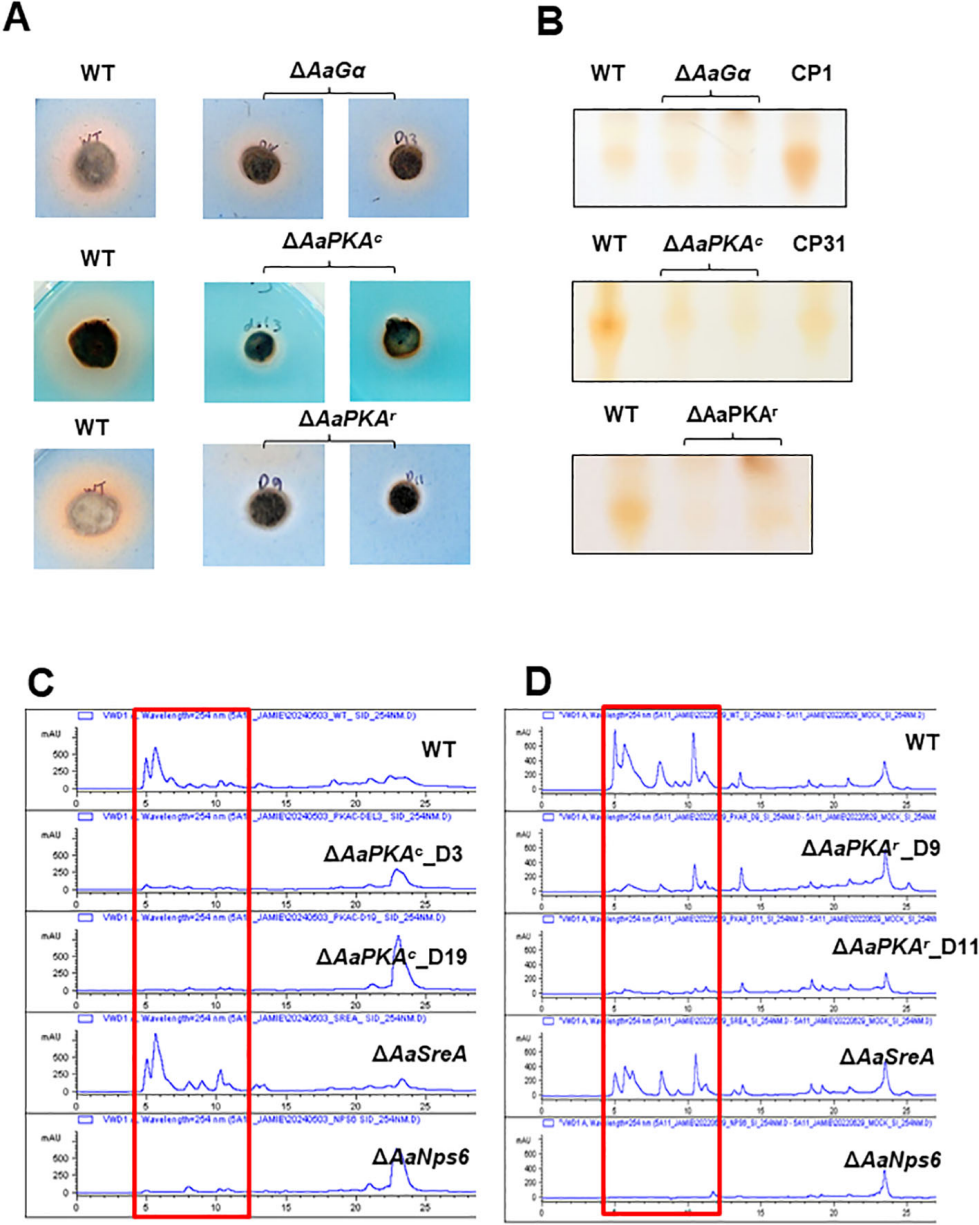
Gα, PKAc, and PKAr contribute to siderophore production. **(A)** CAS plate assays show smaller orange halos in mutant strains compared to WT. **(B)** TLC analysis confirms these observations. **(C, D)** HPLC validates reduced siderophore production in the mutants.

The original version of this article has been updated.

