# Supplementary material for: The adenylate cyclase-mediated signaling pathway required for regulating siderophore and toxin biosynthesis and pathogenicity in Alternaria alternata
Source: Front Fungal Biol. 2026 Feb 5;7:1766476. doi: 10.3389/ffunb.2026.1766476 (PMC12916706; doi:10.3389/ffunb.2026.1766476)
Supplement: Supplementary file 1 [file Supplementaryfile1.docx]

**Supplementary Tables**

**Table S1.** Fungal strains used in the study

| Strain | Description | Reference |
| --- | --- | --- |
| EV-MIL-31 | Wild type | Lin et al., 2009 |
| ∆*AaAC*_27 | *AaAC* deletion strains | This study |
| ∆*AaAC*_30 |  |  |
| CP1 | The AaAC complementation strain | This study |
| ∆∆*AaACTT6* | Double deletions in *ACTT_6-1* and *ACTT_6-2* | Ma et al., 2019 |
| ∆*AaSreA* | Deletion of a siderophore repressor | Chung et al., 2020 |
| ∆*AaNps6* | Deletion of a nonribosomal peptide synthetase | Chen et al., 2013 |
| ∆*AaGα*_D4 | Deletion of a GTP-binding protein alpha subunit | Wang et al., 2010 |
| ∆*AaGα*_D13 |  |  |
| ∆*AaPKA^c^*_D3 | Deletion of a protein kinase A catalytic subunit | Tsai et al., 2013 |
| ∆*AaPKA^c^*_D19 |  |  |
| ∆*AaPKA^r^*_D9 | Deletion of a protein kinase A regulatory subunit |  |
| ∆*AaPKA^r^*_D11 |  |  |
| WT/sGFP-AaAtg8 | Wild-type expressing a sGFP::AaAtg8 fusion protein | This study |
| ∆*AaAC/*sGFP-AaAtg8 | ∆*AaAC* expressing a sGFP::AaAtg8 fusion protein |  |

**Table S2. Oligonucleotides used in the study**

| Primer name | Sequence (5' → 3') | Remarks |
| --- | --- | --- |
| AC-split-F1 | CTCAAATAACCGTCTCCAA | Generation of split marker fragments and confirmation of deletion mutants |
| AC-split-F2 | TGTTTCCTCCAACGTCCT |  |
| AC-split-F | TCCTGTGTGAAATTGTTATCCGCACGATTCACACCACTTTCT |  |
| AC-split-R | GTCGTGACTGGGAAAACCCTGGCGCGGGGTACAACCATATC |  |
| AC-split-R2 | CCCTTTCTTCCTCTGCTT |  |
| AC-split-R1 | GGAAGCAGCGCAGAAAAA |  |
| AC-comp-F_SmaI | AAACCCGGGGGGCCAAAGGATAAGAAA | Genetic complementation |
| AC-comp-R_KpnI | AAAGGTACCCCCTTTCTTCCTCTGCTT |  |
| M13_F | GCCAGGGTTTTCCCAGTCACGAC | Amplification of the Hygromycin resistance gene (*HYG*) and the *HYG* probe |
| M13_R | GCGGATAACAATTTCACACAGGA |  |
| hyg3 | GGATGCCTCCGCTCGAAGTA |  |
| hyg4 | CGTTGCAAGACCTGCCTGAA |  |
| sGFP_F_NEW | ATGGTGAGCAAGGGCGAGGA | Confirmation of Δ*AaAC*/sGFP-AaAtg8 strain |
| Atg8_R_XhoI | ACTCTCGAGCTAGATGGCCTCGCCGAATG |  |
| Tub2Q-1F | TTCGTCGGTAACTCCACCTCCATC | qRT-PCR |
| Tub2Q-1R | ACTCAGCCTCAGTGAACTCCATCTC |  |
| HapX_qPCR_F1 | CGATATGGACTGGCGTCAAC |  |
| HapX_qPCR_R1 | GCTTGCATCTAGCTCCATCG |  |
| SreA_qPCR_F | CTGGTGGAGGGCGATGTA |  |
| SreA_qPCR_R | GAACGCTGGACAACCACT |  |
| Sit1_qPCR_F1 | CGGTATGATTTGCTGCCACA |  |
| Sit1_qPCR_R1 | ACAGGCATCTACCTCGCTAC |  |
| NPR6_qPCR_F | TCGCAACTATTATCCGTGGAG |  |
| NPR6_qPCR_R | CTGGATGGCCCATATACAATG |  |
| ACTT2_qPCR_F | GGATCCGCAAGTACTGGATCTCTT |  |
| ACTT2_qPCR_R | CCTGATATCGTCCTGTGACTAGAC |  |
| ACTT5_qPCR_F | AGCGCATCACAGATCTTGAGACCT |  |
| ACTT5_qPCR_R | AACATGAAACATCGGCAAGGGCGA |  |
| ACTT6_qF | GGTCCATCGTATTGTCCTCAC |  |
| ACTT6_qR | GCATCCTCGTATAGTTCTTCTCC |  |
| AaACTR_qF | AGAACGAGCGACTTCGAG |  |
| AaACTR_qR | CTGTAGACGCAAGGCTGTT |  |

**Table S3.** Differentially expressed genes in the *AaAC* mutant compared to the wild type under nutrient-deficient conditions and their characteristics

| Gene ID | Log2Fold Change | padj | Description |
| --- | --- | --- | --- |
| IG631_12487 | 6.13 | 6.13E-41 | Ornithine aminotransferase |
| IG631_15409 | 2.76 | 2.05E-15 | Two-component signal transduction system (phosphorelay) |
| IG631_10250 | -3.02 | 6.00E-20 | Nonribosomal peptide synthetase dtxS1 |
| IG631_19005 | -3.56 | 3.82E-15 | Siderophore iron transporter mirc |
| IG631_02278 | -0.71 | 0.94 | Acetylornithine aminotransferase |
| IG631_21476 | -1.42 | 4.13E-32 | Siderochrome-iron transporter-like protein Sit1 |
| IG631_21477 | -1.50 | 1.03E-17 | Siderophore iron transporter 1 |
| IG631_12648 | -0.39 | 0.81 | Acetylornithine deacetylase |
| IG631_08034 | -0.68 | 0.15 | Aerobactin siderophore biosynthesis protein iucB |
| IG631_07815 | -2.76 | 0 | Siderophore iron transporter |
| IG631_08875 | -3.14 | 7.79E-42 | Siderophore iron transporter |
| IG631_08874 | -3.28 | 8.38E-09 | Siderophore iron transporter |
| IG631_07801 | -3.16 | 4.39E-147 | Rhizobactin siderophore biosynthesis protein rhbE |
| IG631_02157 | -2.46 | 6.54E-13 | L-ornithine 5-monooxygenase |
| IG631_02156 | -2.38 | 4.63E-76 | L-ornithine |
| IG631_14851 | -2.12 | 1.95E-26 | acetyl-CoA acetyltransferase |
| IG631_02158 | -2.38 | 8.33E-26 | L-ornithine 5-monooxygenase |
| IG631_17402 | -2.28 | 1.79E-18 | Acetylornithine aminotransferase |
| IG631_13121 | -2.48 | 0 | Siderophore iron transporter |
| IG631_09898 | -4.98 | 1.89E-212 | Siderophore iron transporter |
| IG631_07797 | -3.50 | 0 | Nonribosomal peptide synthetase 4 |
| IG631_05488 | 0.02 | 0.19 | Siderophore iron transporter mirB |
| IG631_04434 | -1.58 | 6.85E-28 | Siderophore iron transporter |
| IG631_04433 | -1.48 | 4.02E-05 | Siderophore iron transporter |
| IG631_23057 | 4.58 | 8.68E-08 | Transcription factor (SreA) |
| IG631_04299 | -2.11 | 1.14E-19 | Transcription factor (Hapx) |
| IG631_21158 | -2.25 | 2.14E-62 | Bifunctional acetylglutamate kinase/N-acetyl-gamma-glutamyl-phosphate reductase |
| IG631_19409 | -1.31 | 5.48E-2 | Ornithine carbamoyltransferase |
| IG631_11003 | 2.15 | 1.66E-07 | Arginase |
| IG631_08869 | -2.19 | 4.96E-168 | Ferric reductase |
| IG631_24287 | -3.75 | 1.21E-78 | Polyketide synthase |
| IG631_09397 | -1.51 | 3.34E-30 | Iron transport multicopper oxidase |
| IG631_09396 | -1.59 | 2.41E-24 | Iron transport multicopper oxidase |
| IG631_04572 | -2.96 | 1.76E-15 | ABC-type Fe3+ transport system |
| IG631_19005 | -3.56 | 3.82E-15 | Siderophore iron transporter mirc |
| IG631_10073 | -1.18 | 1.40E-3 | Iron sulfur cluster assembly protein-like protein 1 |
| IG631_15142 | -1.02 | 2.81E-3 | Monothiol glutaredoxin-like protein-4 (GrxD) |
| IG631_19695 | 3.07 | 7.24E-3 | G-alpha-like protein |
| IG631_20673 | -0.61 | 0.13 | S-adenosyl-L-methionine-dependent methyltransferase |
| IG631_01055 | -0.82 | 0.17 | S-adenosyl-L-methionine-dependent methyltransferase |
| IG631_12200 | -1.02 | 0.33 | S-adenosyl-L-methionine-dependent methyltransferase |
| IG631_23129 | -0.63 | 0.79 | Hydroxymethylglutaryl-coa hydrolase |
| IG631_21195 | -0.51 | 0.96 | Hydroxymethylglutaryl-coa hydrolase |
| IG631_02690 | -2.35 | 7.50E-48 | Hydroxymethylglutaryl-coa synthase |
| IG631_24244 | -4.49 | 4.96E-12 | Enoyl-CoA hydratase ACTT6 |
| IG631_24290 | -3.66 | 4.90E-4 | Cytochrome P450 monooxygenase AFT11-1 |
| IG631_07797 | -3.50 | 0 | Nonribosomal peptide synthetase 4 |
| IG631_24152 | -4.60 | 1.29E-58 | Nonribosomal peptide synthetase ACTTS4 |
| IG631_24211 | -1.53 | 1.67E-18 | Enoyl-CoA hydratase ACTT3 |
| IG631_24212 | -1.17 | 1.40E-3 | Enoyl-CoA hydratase ACTT3 |
| IG631_08222 | 1.91 | 1.19E-54 | Autophagy-related protein 13 |
| IG631_05876 | 2.085 | 5.34E-19 | Autophagy-related protein 3 |
| IG631_05877 | 2.31 | 5.40E-17 | Autophagocytosis protein |
| IG631_03386 | -1.76 | 1.39E-3 | autophagy-related protein 22-1 |
| IG631_08221 | 2.06 | 6.92E-3 | Autophagy-related protein 13 |
| IG631_07050 | 1.22 | 1.90E-10 | ER-derived vesicles protein ERV14 |
