# Supplementary material for: The adenylate cyclase-mediated signaling pathway required for regulating siderophore and toxin biosynthesis and pathogenicity in Alternaria alternata
Source: Front Fungal Biol. 2026 Feb 5;7:1766476. doi: 10.3389/ffunb.2026.1766476 (PMC12916706; doi:10.3389/ffunb.2026.1766476)
Supplement: Supplementary file 2 [file Supplementaryfile2.pdf]

Supplementary Material

Figure S1

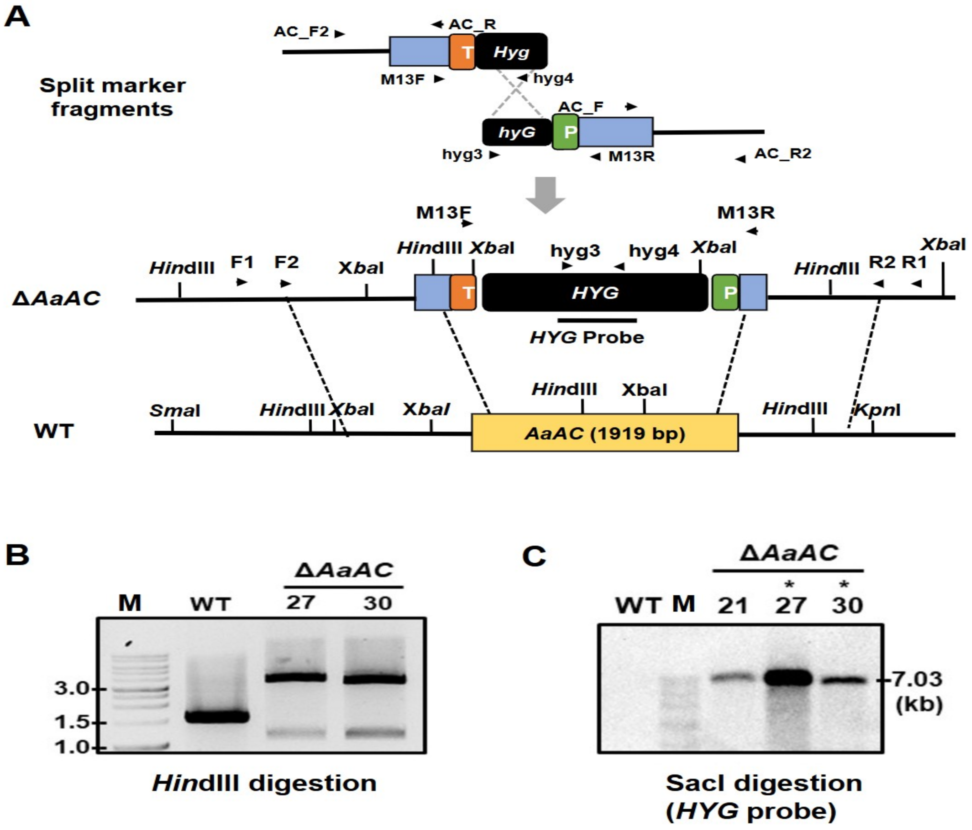

Supplementary FIGURE S1. Genetic modification and complementation of *AaAC*. (A) Schematic showing *AaAC* replacement with *HYG* using the split-marker approach. The 5′ and 3′ ends of *AaAC* (1919 bp) were amplified and fused with overlapping *HYG* fragments to generate split-marker constructs. (B) RFLP analysis confirming Δ*AaAC*. PCR products amplified with AC_split_F2/AC_split_R2 were digested with HindIII and resolved on a 1% agarose gel. (C) Southern blot hybridization of fungal DNA, after SacI digestion, with a *HYG* probe detects a 7,036 bp band in Δ*AaAC* mutants but not in wild-type (WT). M: size marker.

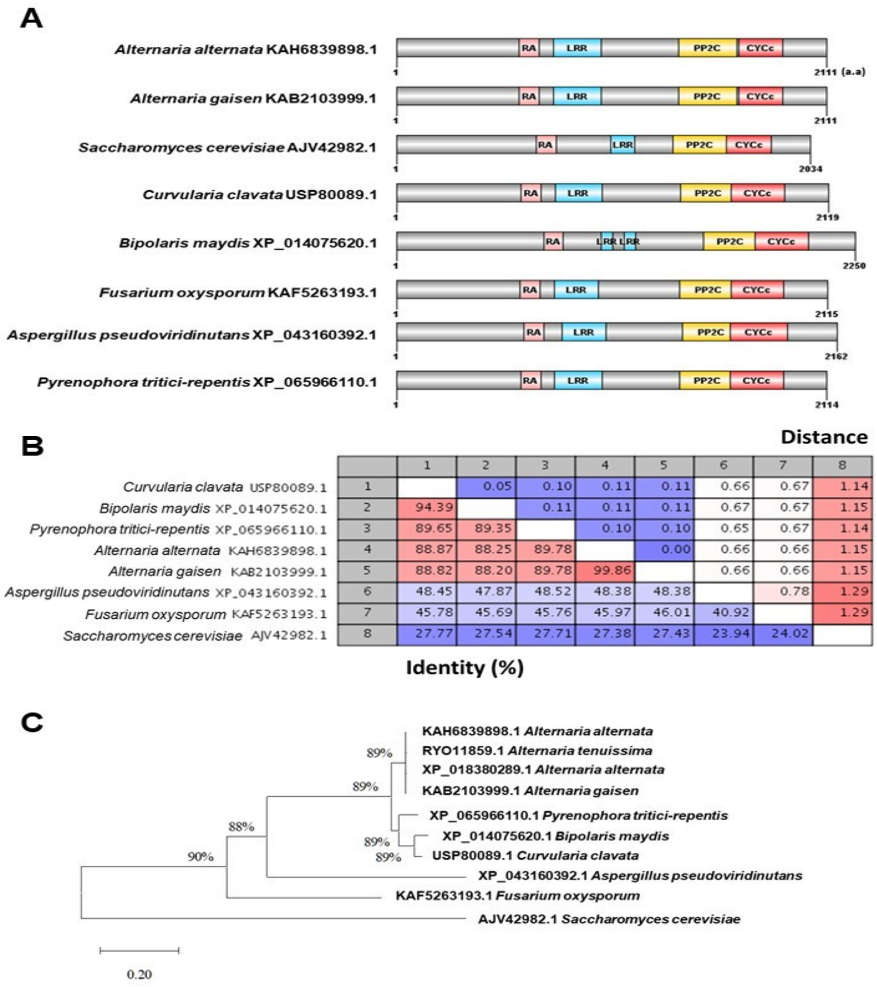

Supplementary FIGURE S2. Characterization of adenylate cyclase in fungi. (A) Protein motifs of adenylate cyclase in fungi identified using InterProScan and NCBI CDART**.** Abbreviations: RA: Ras-associating domain; LRR: Leucine-rich repeat protein; PP2C: Protein phosphatase 2C; and CYCc: Adenylate/guanylate cyclase catalytic domain. (B) Pairwise sequence comparisons of fungal adenylate cyclase. (C) Phylogenetic analysis reveals that AaAC shared ~90% similarity with other phytopathogenic fungi.

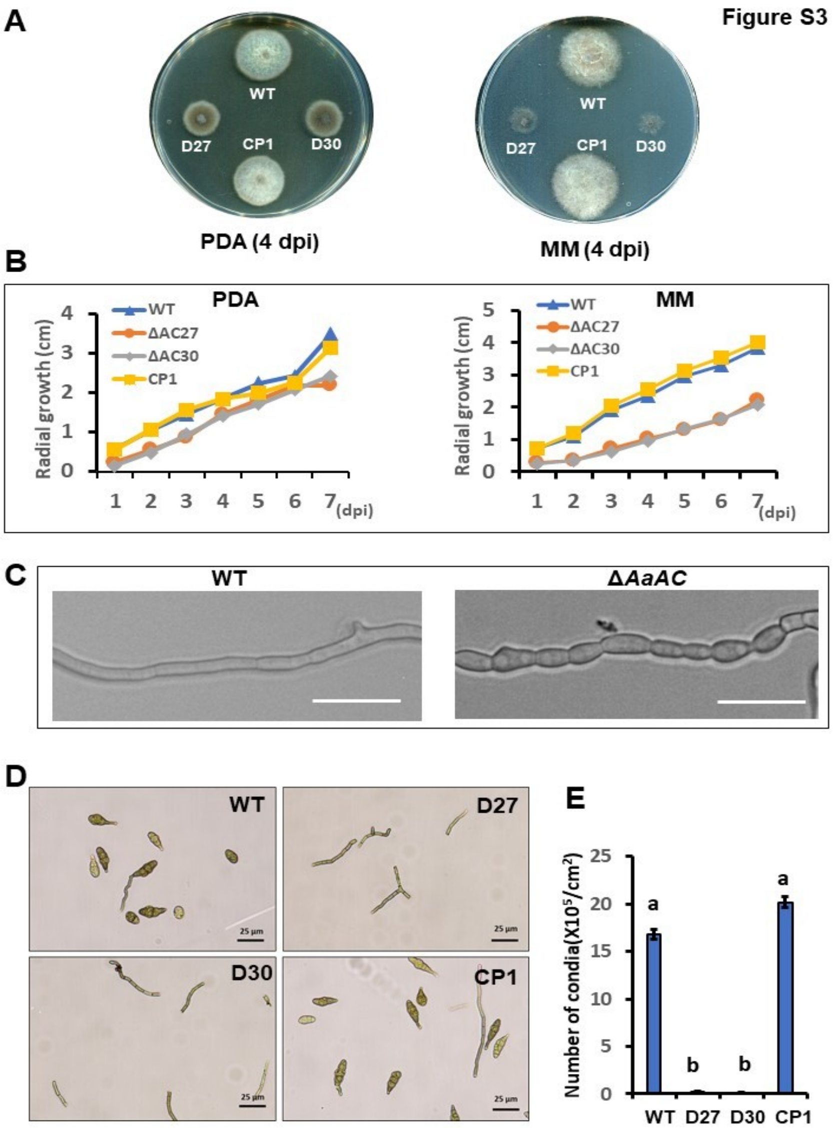

Supplementary FIGURE S3. AaAC is essential for vegetative growth, differentiation, and conidiation. (A) Mycelial growth of WT, Δ*AaAC*, and CP1 on PDA and MM media. (B) Growth curves of WT, Δ*AaAC*, and CP1 over 7 days. (C) Deletion of *AaAC* leads to hyphal deformation. (D) Conidial morphology of WT, Δ*AaAC*, and CP1. Scale bar = 25 μm. (E) Quantitative analysis of conidia production by fungal strains grown on PDA. Means followed by the same letters are not significantly different, $p < 0.05$.

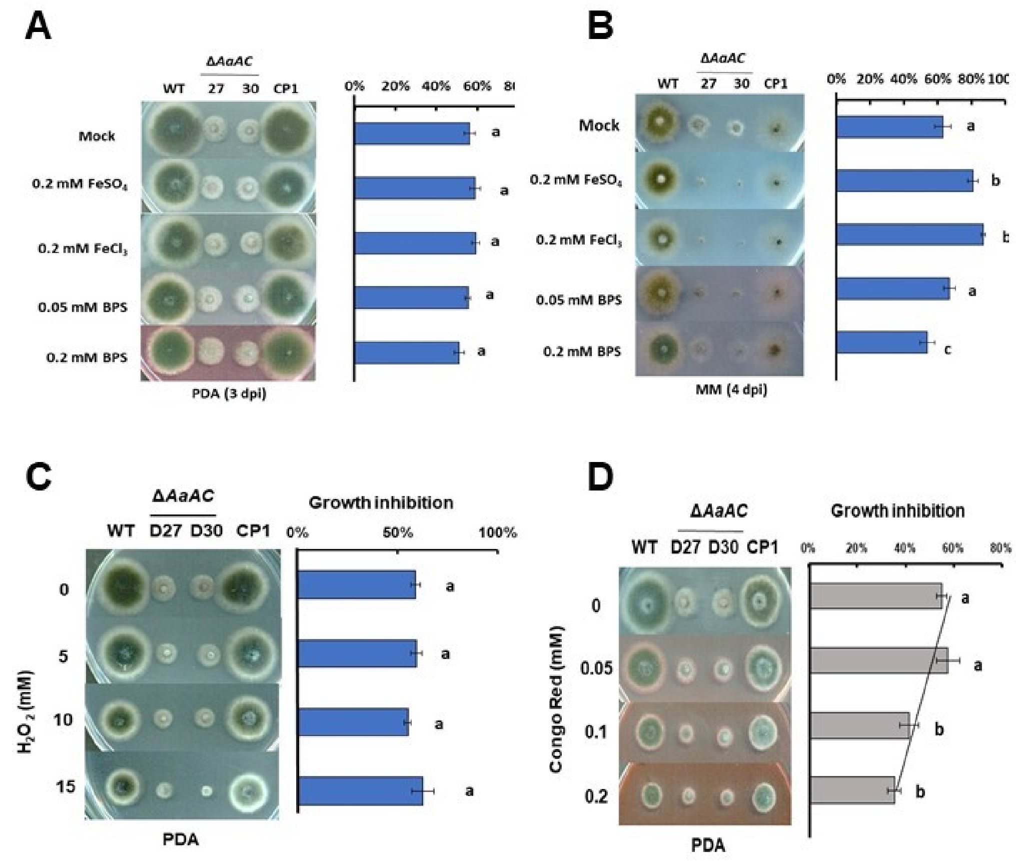

Supplementary FIGURE S4. AaAC contributes to iron homeostasis and cell wall integrity but is dispensable for H_2O_2 resistance. (A) (B) Sensitivity assays of WT, Δ*AaAC*, and CP1 strains grown on PDA or MM supplemented with 0.5 mM $FeCl_3$, 0.5 mM $FeSO_4$, or 0.05–0.2 mM bathophenanthroline disulfonic acid (BPS). (C) The Δ*AaAC* mutant displays no changes in sensitivity to H_2O_2. (D) The Δ*AaAC* mutant exhibits reduced sensitivity to Congo red in a dose-dependent manner. The percentage changes in radial growth was calculated as the percentage of growth of the mutant strains compared to WT grown on the same plate. Means followed by the same letters are not significantly different, $p < 0.05$.
